# Retraction Note: Inhibition of PAD4 enhances radiosensitivity and inhibits aggressive phenotypes of nasopharyngeal carcinoma cells

**DOI:** 10.1186/s11658-024-00683-6

**Published:** 2024-12-30

**Authors:** Hao Chen, Min Luo, Xiangping Wang, Ting Liang, Chaoyuan Huang, Changjie Huang, Lining Wei

**Affiliations:** 1 Department of Endoscopy, The Affiliated Tumor Hospital of Guangxi Medical University, Nanning, 530021 Guangxi China; 2 Department of Oncology, The Second Nanning People’s Hospital, Jiangnan District, No.13 Dancun Road, Nanning, 530031 Guangxi China

**Retraction Note: Cellular & Molecular Biology Letters (2021) 26:9** 10.1186/s11658-021-00251-2

The Editor-in-Chief has retracted this article because of concerns regarding the soundness of the data presented. After publication, the authors informed the journal that the invasion and Western blot data reported in this work are incorrect and that they had not personally conducted these experiments. In light of this and previous concerns involving figures presented in this article, the Editor-in-Chief therefore no longer has confidence in the results and conclusions presented in this article.

The authors agree with this retraction.

